# Effects of *Kt*/*V*_urea_ on outcomes according to age in patients on maintenance hemodialysis

**DOI:** 10.1093/ckj/sfae116

**Published:** 2024-04-13

**Authors:** Junseok Jeon, Gui Ok Kim, Bo Yeon Kim, Eun Jung Son, Jun Young Do, Jung Eun Lee, Seok Hui Kang

**Affiliations:** Division of Nephrology, Department of Medicine, Samsung Medical Center, Sungkyunkwan University School of Medicine, Seoul, Republic of Korea; Health Insurance Review and Assessment Service, Wonju, Republic of Korea; Health Insurance Review and Assessment Service, Wonju, Republic of Korea; Health Insurance Review and Assessment Service, Wonju, Republic of Korea; Division of Nephrology, Department of Internal Medicine, College of Medicine, Yeungnam University, Daegu, Republic of Korea; Division of Nephrology, Department of Medicine, Samsung Medical Center, Sungkyunkwan University School of Medicine, Seoul, Republic of Korea; Division of Nephrology, Department of Internal Medicine, College of Medicine, Yeungnam University, Daegu, Republic of Korea

**Keywords:** dialysis dose, elderly, hemodialysis, Kt/V, mortality, outcome

## Abstract

**Background:**

The guidelines recommended target and minimum single-pool *Kt*/*V*_urea_ are 1.4 and 1.2, respectively, in hemodialysis patients. However, the optimal hemodialysis dose remains controversial. We investigated the effects of *Kt*/*V*_urea_ on patient outcomes according to age, with a focus on older patients.

**Methods:**

This study used the hemodialysis quality assessment program and claims datasets. Patients were divided into four subgroups according to age (<65, 65–74, 75–84, and ≥85 years). Each group was divided into three subgroups according to *Kt*/*V*_urea_ : reference (ref) (1.2 ≤ *Kt*/*V*_urea_ ≤ 1.4), low (< 1.2), and high (> 1.4).

**Results:**

The low, ref, and high *Kt*/*V*_urea_ groups included 1668, 8156, and 16 546 (< 65 years); 474, 3058, and 7646 (65–74 years); 225, 1362, and 4194 (75–84 years); and 14, 126, and 455 (≥85 years) patients, respectively. The low *Kt*/*V*_urea_ group had higher mortality rates than the ref *Kt*/*V*_urea_ group irrespective of age [adjusted hazard ratio (aHR), 95% confidence interval (CI): 1.23, 1.11–1.36; 1.14, 1.00–1.30; 1.28, 1.09–1.52; and 2.10, 1.16–3.98, in patients aged <65, 65–74, 75–84, and ≥85 years, respectively]. The high *Kt*/*V*_urea_ group had lower mortality rates than the ref *Kt*/*V*_urea_ group in patients aged <65 and 65–74 years (aHR, 95% Cl: 0.87, 0.82–0.92 and 0.93, 0.87–0.99 in patients aged <65 and 65–74 years, respectively).

**Conclusions:**

These results support the current recommendations of a minimum *Kt*/*V*_urea_ of 1.2 even in patients age ≥85 years. In young patients, *Kt*/*V*_urea_ above the recommended threshold can be beneficial for survival.

KEY LEARNING POINTS **What was known:**

- The current recommended target and minimum single-pool *Kt*/*V*_urea_ values are 1.4 and 1.2, respectively, in patients undergoing hemodialysis (HD). However, there is limited data on the association between HD adequacy and outcomes, particularly in elderly patients.

**This study adds:**

- We investigated the effects of *Kt*/*V*_urea_ on patient outcomes according to age. The findings support the current recommendations of maintaining a minimum *Kt*/*V*_urea_ of 1.2, even in patients aged 85 years and above. In younger patients, achieving a *Kt*/*V*_urea_ above the recommended threshold can be beneficial for survival.

**Potential impact:**

- Maintaining a certain level of adequacy in HD, even in elderly patients, is deemed helpful. In younger patients, it is anticipated that maintaining HD above the recommended threshold, if tolerable, would be beneficial.

## INTRODUCTION

The prevalence of end-stage kidney disease (ESKD) is increasing worldwide, especially in people aged 75 years and older [1, 2]. In the Republic of Korea, patients aged 65 years or older have accounted for >50% of patients with ESKD since 2019 [3]. However, whether dialysis can prolong survival in older patients with comorbidities, especially ischemic heart disease, remains unanswered [4]. Moreover, dialysis often does not appear to improve the overall health of older patients [5]. Given that a significant proportion of older patients with ESKD are not candidates for kidney transplantation [6], providing them with the appropriate dialysis care to improve their quality of life and survival is a crucial issue.

Urea *Kt*/*V* [*Kt*/*V*_urea_, a product of dialyzer urea clearance (*K*) and treatment time (*t*) divided by urea distribution volume (*V*)] is the preferred assessment of the delivered dose of dialysis. Furthermore, *Kt*/*V*_urea_ is the most basic indicator of dialysis adequacy, and clinical guidelines for hemodialysis (HD) recommend a target single-pool *Kt*/*V*_urea_ (sp*Kt*/*V*_urea_) of 1.4 with a minimum of 1.2 [7] or equilibrated *Kt*/*V*_urea_ of 1.2 (equivalent to sp*Kt*/*V*_urea_ of 1.4) [8]. However, the randomized controlled trial that this recommendation was based on, had excluded older patients and those with comorbidities [9, 10]. Using *Kt*/*V*_urea_ as an indicator of dialysis adequacy is intended to prevent uremic complications, but *Kt*/*V*_urea_ has not been adequately validated in older patients. In contrast, maintaining a high dose of dialysis requires a high blood flow rate, a dialyzer with a large surface area, or a long dialysis time, which requires more frequent vascular interventions, exposing patients to dialysis disequilibrium, and a decrease in patient quality of life [11, 12].

Few studies have focused on the role of *Kt*/*V*_urea_ in the older population [13]. Although the Hemodialysis (HEMO) study, a landmark trial on the relationship between *Kt*/*V*_urea_ and mortality, included patients up to the age of 80 years, the average age of patients in the study was ∼56 years [14]. Therefore, whether *Kt*/*V*_urea_ is an appropriate parameter for HD adequacy in the older population continues to be debated [15]. Here, we investigated the association between *Kt*/*V*_urea_ and patient outcomes according to age with a focus on older patients on maintenance HD in a large-scale nationwide cohort.

## MATERIALS AND METHODS

### Data source and study population

In the Republic of Korea, regular HD quality assessment programs are conducted to ensure quality control [16]. The fourth (July and December 2013), fifth (July and December 2015), and sixth (March and August 2018) HD quality assessment programs were conducted for adult patients (age ≥18 years) who have been receiving maintenance HD for a minimum of 3 months and at least twice per week. Briefly, data for the HD quality assessment program was collected as follows: Health Insurance Review and Assessment Service (HIRA) announced the implementation plans and duration of each program (6 months) in the respective HD centers several months ago before patient recruitment. The data collection targeted all patients treated at the hospitals performing HD. For outpatients, patients were excluded if they were hospitalized during the 6-month period, received less than twice per week or fewer than eight sessions of HD per month, and discontinued outpatient visits. For inpatients, the list was provided to each hospital for all patients, excluding those with less than eight HD sessions per month or those whose hospitalizations were discontinued during the specified period. Among them, HIRA provided each hospital with a list of randomly selected patients to input their data. Each hospital was then responsible for entering the data for the respective patients over the 6-month period monthly. We retrospectively analyzed the data collected by the HIRA during HD quality assessment programs as well as claims and death data.

**Figure 1:**
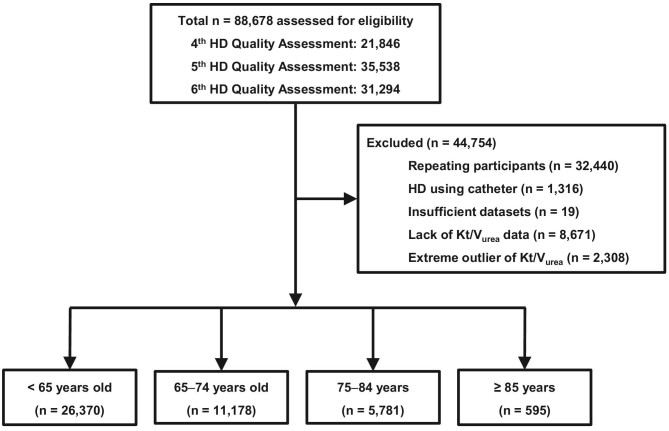
Study flowchart.

The study's schematic representation is presented in Fig. 1. From an initial pool of 88 678 participants in the fourth, fifth, and sixth HD quality assessment programs, we included only the first of multiple participations and excluded participants who underwent HD with a catheter and those with insufficient data, without *Kt*/*V*_urea_ assessments, and with extreme *Kt*/*V*_urea_ values (≤2.5 or 97.5th ≥ percentile). This resulted in a study cohort of 43 924 patients.

### Exposure

*Kt*/*V*_urea_ was computed using the Daugirdas equation [17] as follows: *Kt*/*V*_urea_ = −ln(*R* − 0.008 × *t*) + (4−3.5 × *R*) × UFV/*W* [ln, natural logarithm; *R*, postdialysis blood urea nitrogen (BUN)/predialysis BUN; *t*, time; UFV, ultrafiltration volume; *W*, postdialysis body weight]. Additionally, HD quality assessment programs collected data on *Kt*/*V*_urea_ for 6 months, and patients with values ≥1 *Kt*/*V*_urea_ were identified. We used the average of these assessments collected monthly and divided the patients into four groups according to age (<65, 65–74, 75–84, or ≥85 years) including those who were 85 years and older to account for the increasing number of patients on dialysis in older age groups [18, 19]. In each group, we further divided people into three groups using minimal or target values of *Kt*/*V*_urea_ according to previous guidelines [7]: reference (ref) *Kt*/*V*_urea_ group, 1.2 ≤  *Kt*/*V*_urea_ ≤ 1.4; low *Kt*/*V*_urea_ group, *Kt*/*V*_urea_ < 1.2; and high *Kt*/*V*_urea_ group, *Kt*/*V*_urea_ > 1.4.

### Study variables

We compiled the information on several variables, such as age, sex, HD vintage (months), underlying etiology of ESKD, and the type of vascular access used. Additionally, several clinical parameters were documented during the evaluation, which included measurements of blood hemoglobin levels (g/dl); serum levels of albumin (g/dl), calcium (mg/dl), phosphorus (mg/dl), and creatinine (mg/dl); and ultrafiltration volume (l/session). The data were collected monthly, and all laboratory values were derived as averages from these monthly datasets.

Medications, such as anti-hypertensive drugs, aspirin, clopidogrel, and statins were evaluated using the medication codes provided in Table S1. The use of medication was defined as one or more prescriptions identified during an HD quality assessment program. Before the HD quality assessments, comorbidities were evaluated over a year. Comorbidities were defined using the Charlson comorbidity index (CCI), which encompasses 17 different comorbid conditions (Table S2) [20, 21]. Furthermore, CCI scores were computed for all patients. Additionally, the presence of myocardial infarction or congestive heart failure was identified based on ICD-10 codes.

### Outcomes

Patients were followed up until April 2022. We evaluated all-cause mortality as the primary outcome and cardiovascular events (CVEs) as the secondary outcome. The ICD-10 codes and medical treatments, procedures, or operation codes were used to evaluate CVEs (Table S3). The incidence of death and death date were collected from the HIRA. If a patient was transferred to peritoneal dialysis or kidney transplantation without an event, it was considered censored at that time.

### Statistical analyses

The data were analyzed using two statistical software packages: SAS Enterprise Guide v.7.1 and R v.3.5.1. Categorical variables are presented as frequencies and percentages, whereas continuous variables are presented as means and standard deviations. The statistical significance of differences between categorical variables was assessed using Pearson's *χ*^2^ test or Fisher's exact test. Differences between continuous variables were assessed using a one-way analysis of variance followed by Tukey's *post hoc* test.

Survival curves were estimated using the Kaplan–Meier curves. *P* values for the comparison of survival curves were determined using the log-rank test. Hazard ratio (HR) and confidence interval (CI) were calculated using Cox regression analyses. Multivariable Cox regression analyses were adjusted for age; sex; body mass index; vascular access type; CCI score; HD vintage; ultrafiltration volume; blood levels of hemoglobin, albumin, creatinine, phosphorus, and calcium; use of anti-hypertensive drugs, aspirin, clopidogrel, or statins; and myocardial infarction or congestive heart failure. Multivariable Cox regression analyses were performed using the enter mode. Statistical significance was set at a two-tailed *P* < 0.05.

### Statement of ethics

The research protocol was approved by the institutional review board of Yeungnam University Medical Center (approval no. YUMC 2022–01-010). Informed consent was not required as patient records and information had been anonymized and de-identified before the analysis.

## RESULTS

### Baseline characteristics

The numbers of patients aged <65, 65–74, 75–84, and ≥85 years were 26 370, 11 178, 5781, and 595, respectively (Table 1). Compared to other age groups, patients aged <65 years included higher proportions of male sex and autologous arteriovenous fistula and lower proportions of the use of aspirin, clopidogrel, statins, and myocardial infarction or congestive heart failure. Moreover, patients aged <65 years exhibited elevated levels of blood calcium, phosphorus, creatinine, hemoglobin, and albumin, along with increased ultrafiltration volume, and lower CCI scores compared to other age groups. Among the age groups, the highest prevalence of diabetic nephropathy was observed in patients aged 66–74 years. The overall numbers of patients in the low, ref, and high *Kt*/*V*_urea_ groups wrtr 1668, 8156, and 16 546 patients aged <65 years, respectively; 474, 3058, and 7646 in patients aged 65–74 years, respectively; 225, 1362, and 4194 in patients aged 75–84 years, respectively; and 14, 126, and 455 patients aged ≥85 years, respectively.

**Table 1:** Clinical characteristics of the patients.

	<65 years old (*n* = 26 370)	66–74 years old (*n* = 11 178)	75–84 years old (*n* = 5781)	≥85 years old (*n* = 595)	*P*
Age (years)	51.9 ± 9.0	69.4 ± 2.9*	78.3 ± 2.6^*#^	87.0 ± 2.3^*#+^	<.001
Sex (male, %)	16 195 (61.4)	6552 (58.6)	3282 (56.8)	314 (52.8)	<.001
Hemodialysis vintage (months)	55 ± 60	48 ± 50*	39 ± 41^*#^	39 ± 38^*#^	<.001
Body mass index (kg/m^2^)	22.6 ± 3.0	22.6 ± 3.2	22.2 ± 3.1^*#^	21.7 ± 3.1	<.001
Underlying causes of ESKD					<.001
Diabetes mellitus	10 801 (41.0%)	5915 (52.9%)	2666 (46.1%)	212 (35.6%)	
Hypertension	6406 (24.3%)	2916 (26.1%)	1874 (32.4%)	238 (40.0%)	
Glomerulonephritis	3585 (13.6%)	701 (6.3%)	300 (5.2%)	22 (3.7%)	
Others	2569 (9.7%)	713 (6.4%)	361 (6.2%)	46 (7.7%)	
Unknown	3009 (11.4%)	933 (8.3%)	580 (10.0%)	77 (12.9%)	
CCI score	7.0 ± 2.8	8.3 ± 2.8*	8.4 ± 2.8^*#^	8.4 ± 2.8*	<.001
Autologous arteriovenous fistula	23 234 (88.1%)	9240 (82.7%)	4480 (77.5%)	428 (71.9%)	<.001
*Kt*/*V*_urea_	1.51 ± 0.23	1.53 ± 0.22*	1.56 ± 0.22^*#^	1.58 ± 0.22^*#+^	<.001
Ultrafiltration volume (l/session)	2.46 ± 0.95	2.09 ± 0.88*	1.89 ± 0.90^*#^	1.70 ± 0.81^*#+^	<.001
Hemoglobin (g/dl)	10.7 ± 0.8	10.6 ± 0.7*	10.6 ± 0.7*	10.6 ± 0.7*	<.001
Serum albumin (g/dl)	4.05 ± 0.33	3.92 ± 0.33*	3.84 ± 0.33^*#^	3.76 ± 0.34^*#+^	<.001
Serum phosphorus (mg/dl)	5.26 ± 1.39	4.60 ± 1.18*	4.35 ± 1.12^*#^	4.15 ± 1.02^*#+^	<.001
Serum calcium (mg/dl)	8.95 ± 0.83	8.83 ± 0.81*	8.75 ± 0.76^*#^	8.73 ± 0.75^*#^	<.001
Serum creatinine (mg/dl)	10.3 ± 2.7	8.6 ± 2.3*	7.6 ± 2.2^*#^	6.9 ± 2.0^*#+^	<.001
Use of anti-hypertensive drugs	18 078 (68.6%)	8080 (72.3%)	3888 (67.3%)	356 (59.8%)	<.001
Use of aspirin	10 772 (40.8%)	5364 (48.0%)	2668 (46.2%)	264 (44.4%)	<.001
Use of clopidogrel	3651 (13.8%)	2436 (21.8%)	1311 (22.7%)	110 (18.5%)	<.001
Use of statins	7635 (29.0%)	4074 (36.4%)	2017 (34.9%)	177 (29.7%)	<.001
MI or CHF	10 916 (41.4%)	5810 (52.0%)	3216 (55.6%)	344 (57.8%)	<.001

Data are expressed as mean ± standard deviation for continuous variables and numbers (percentages) for categorical variables. *P* values were tested using a one-way analysis of variance, followed by Tukey’s *post hoc* test, and Pearson's *χ*^2^ test for categorical variables.

**Abbreviations:** CCI, Charlson Comorbidity Index; CHF, congestive heart failure; ESKD, end-stage kidney disease; MI, myocardial infarction. *P < 0.05 vs. <65 years of age; ^#^*P* < 0.05 vs. 66–74 years of age; ^+^*P* < 0.05 vs. 75–84 years of age.

### Overall survival according to *Kt*/*V*_urea_

**Figure 2:**
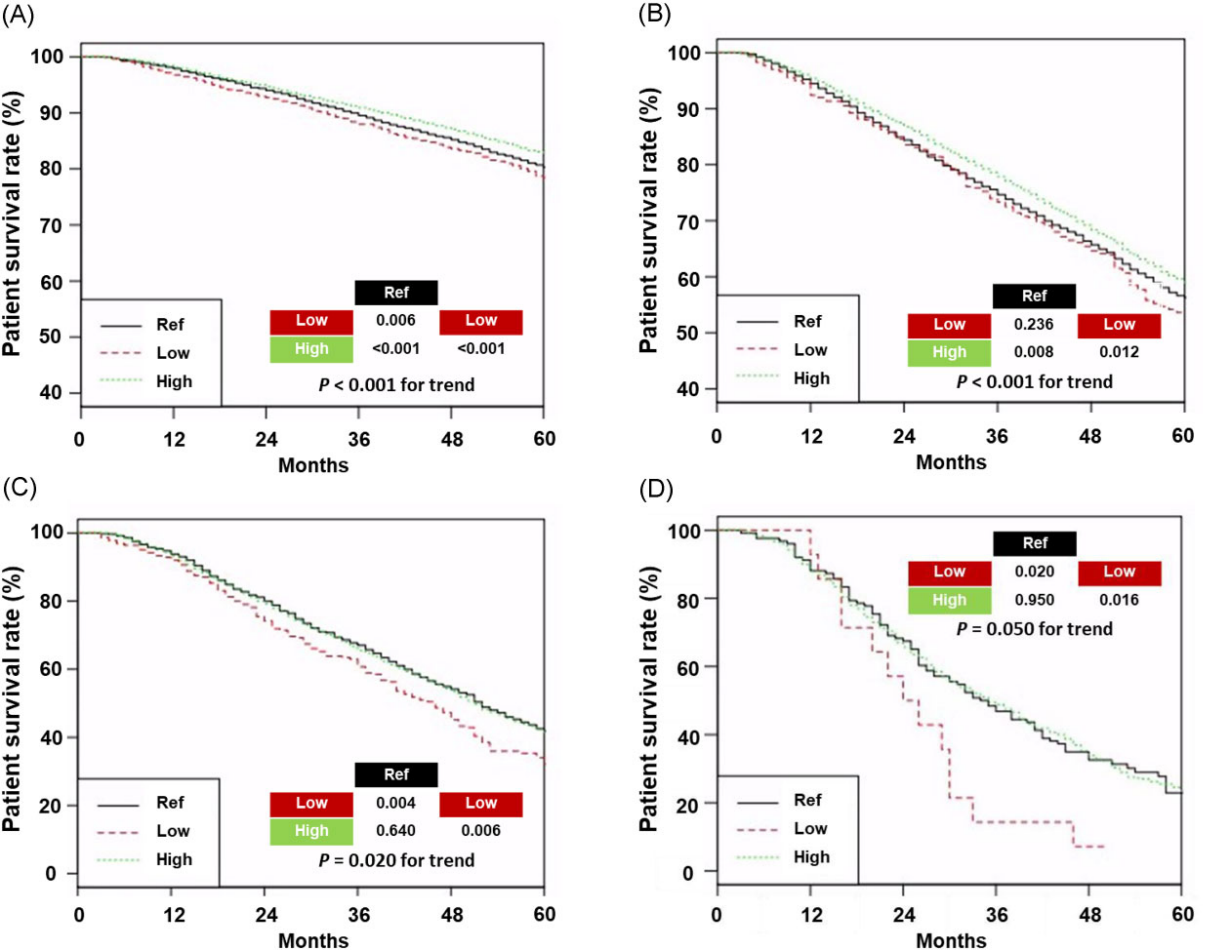
Kaplan–Meier curves of patient survival based on groups across different age categories. Survival curves in patients aged <65 years (A), 65–74 years (B), 75–84 years (C), and ≥85 years. Abbreviations: Ref group: patients with 1.2 ≤ *Kt*/*V*_urea_ ≤ 1.4; low group, patients with *Kt*/*V*_urea_ < 1.2; high group, patients with *Kt*/*V*_urea_ > 1.4.

The follow-up duration was 66 ± 28 months in patients aged <65 years, 57 ± 28 months in patients aged 65–74 years, 48 ± 25 months in patients aged 75–84 years, and 37 ± 21 months in patients aged ≥85 years. The 5-year survival rate in the low, ref, and high *Kt*/*V*_urea_ groups was 78.4%, 80.3%, and 82.7% in patients aged <65 years, respectively; 53.6%, 56.3%, and 59.0% in patients aged 65–74 years, respectively; 32.2%, 41.8%, and 41.4% in patients aged 75–84 years, respectively; and 7%, 22.9%, and 23.5% in patients aged ≥85 years, respectively (Fig. 2).

In patients aged <65 years, the high *Kt*/*V*_urea_ group had the best patient survival rate among the three groups, and the ref *Kt*/*V*_urea_ group had a better patient survival rate than the low *Kt*/*V*_urea_ group. In those aged 65–74 years, the high *Kt*/*V*_urea_ group had a better patient survival rate than the other groups. In patients aged 75–84 or ≥85 years, the low *Kt*/*V*_urea_ group had the worst patient survival rate among the three groups.

Table 2 presents the results of the Cox regression analyses. Univariable and multivariable Cox regression analyses using patients aged <65 years demonstrated similar trends with those from the Kaplan–Meier curves. Multivariable Cox regression analyses for different age groups revealed that the low *Kt*/*V*_urea_ group exhibited the poorest patient survival compared to the other two groups.

**Table 2:** *Kt*/*V*_urea_ and the associated risk of all-cause mortality and cardiovascular events according to age groups.

	All-cause mortality				CVEs			
	Univariable		Multivariable		Univariable		Multivariable	
	HR (95% CI)	*P*	HR (95% CI)	*P*	HR (95% CI)	*P*	HR (95% CI)	*P*
<65 years old								
Ref								
Low	1.15 (1.04–1.27)	.006	1.23 (1.11–1.36)	<.001	1.09 (0.96–1.25)	.193	1.11 (0.96–1.28)	.152
High	0.87 (0.87–0.91)	<.001	0.87 (0.82–0.92)	<.001	0.91 (0.84–0.97)	.007	0.95 (0.88–1.03)	.202
Low								
High	0.75 (0.69–0.83)	<.001	0.71 (0.64–0.78)	<.001	0.83 (0.73–0.94)	.004	0.86 (0.75–0.99)	.030
65–74 years old								
Ref								
Low	1.08 (0.95–1.22)	.234	1.14 (1.00–1.30)	.044	1.01 (0.81–1.26)	.934	1.00 (0.79–1.25)	.988
High	0.92 (0.87–0.97)	.003	0.93 (0.87–0.99)	.031	0.94 (0.85–1.03)	.189	1.02 (0.92–1.14)	.686
Ref. Low								
High	0.85 (0.75–0.96)	.008	0.82 (0.72–0.93)	.002	0.93 (0.75–1.15)	.492	1.02 (0.82–1.28)	.834
75–84 years old								
Ref								
Low	1.27 (1.07–1.49)	.005	1.28 (1.09–1.52)	.003	1.03 (0.73–1.47)	.867	1.03 (0.72–1.48)	.879
High	1.02 (0.94–1.10)	.647	0.98 (0.91–1.06)	.647	0.97 (0.84–1.13)	.714	0.99 (0.84–1.17)	.929
Ref Low								
High	0.80 (0.69–0.94)	.006	0.77 (0.65–0.90)	.001	0.94 (0.67–1.32)	.734	0.97 (0.68–1.37)	.843
≥85 years old								
Ref								
Low	1.99 (1.11–3.54)	.020	2.10 (1.16–3.81)	.014	0.80 (0.10–6.08)	.828	1.04 (0.13–8.14)	.974
High	1.01 (0.81–1.25)	.948	0.90 (0.71–1.14)	.393	1.44 (0.81–2.56)	.213	1.48 (0.78–2.83)	.233
Ref. Low								
High	0.51 (0.29–0.88)	.017	0.43 (0.24–0.77)	.004	1.80 (0.25–13.02)	.558	1.43 (0.19–10.74)	.727

Multivariable analysis was adjusted for age, sex, body mass index, vascular access type, hemodialysis vintage, charlson comorbidity index score, ultrafiltration volume, hemoglobin, serum albumin, serum creatinine, serum phosphorus, serum calcium, use of anti-hypertensive drugs, statin, clopidogrel, or aspirin, and myocardial infarction or congestive heart failure, and was performed using enter mode.

### CVE-free survival according to *Kt*/*V*_urea_

**Figure 3:**
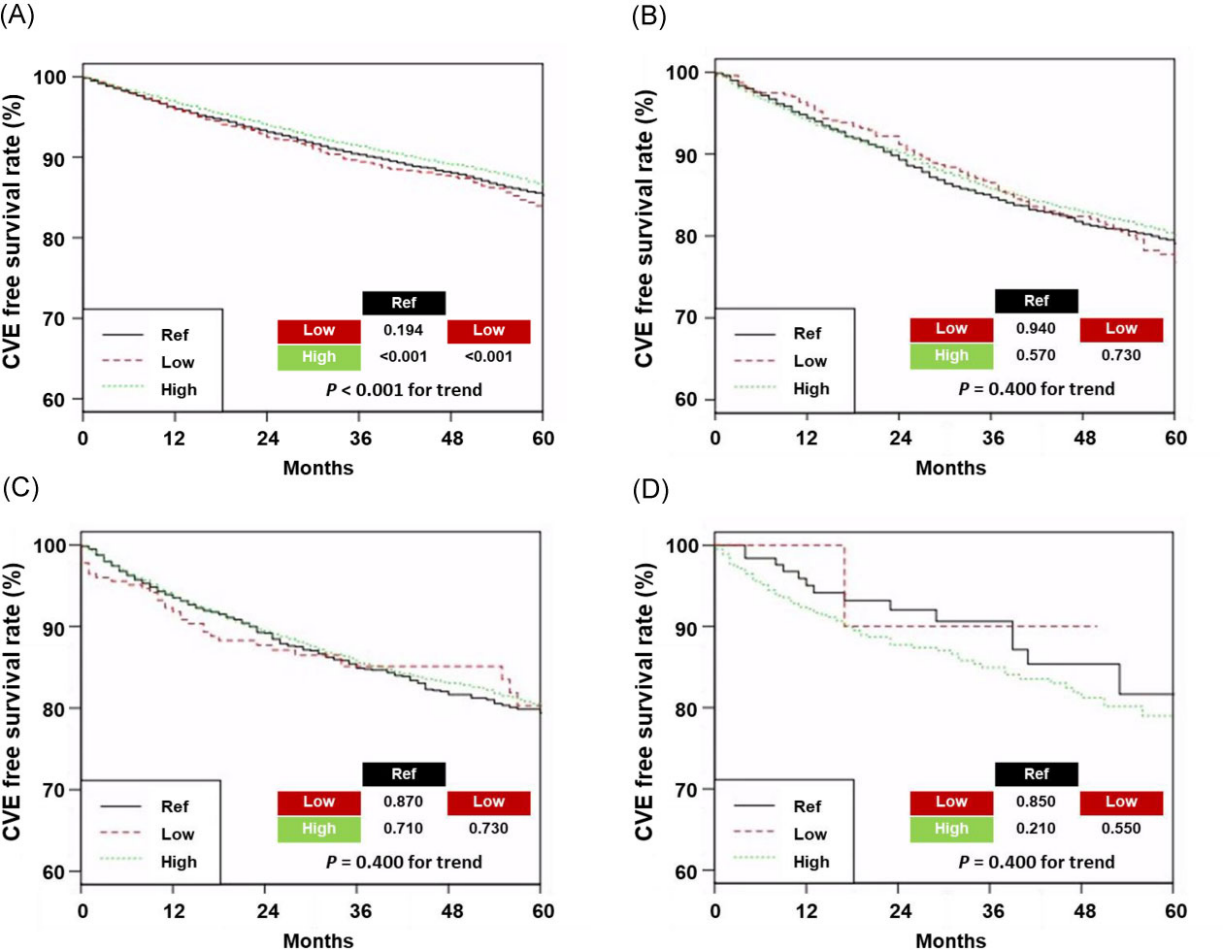
Kaplan–Meier curves of cardiovascular events (CVEs) according to *Kt*/*V*_urea_ across different age groups. Patient survival curves in <65 years (A), 65–74 years (B), 75–84 years (C), and ≥85 years of age.

The 5-year CVE-free survival rate in the low, ref, and high *Kt*/*V*_urea_ groups was 83.9%, 85.3%, and 86.6% in patients aged <65 years, respectively; 76.8%, 77.3%, and 80.1% in patients aged 65–74 years, respectively; 80.3%, 79.4%, and 80.2% in patients aged 75–84 years, respectively; and 90%, 82%, and 79% in patients aged ≥85 years, respectively (Fig. 3). In those aged <65 years, the high *Kt*/*V*_urea_ group had the best CVE-free survival rate among the three groups. On multivariable Cox regression analyses, no statistically significant differences were observed between the *Kt*/*V*_urea_ groups and CVE in all age groups except between the low and high *Kt*/*V*_urea_ groups in patients aged <65 years (Table 2).

### Relationship between *Kt*/*V*_urea_ and HR

Figure S1 presents the spline curves depicting the relationship between *Kt*/*V*_urea_ and HR for patient mortality. In patients aged <65, 65–74, and 75–84 years, both unadjusted and adjusted spline curves indicated improved patient survival with high *Kt*/*V*_urea_ values and diminished patient survival with low *Kt*/*V*_urea_ values. However, in patients aged ≥85 years, the distinct trend was diminished.

Figure S2 presents the spline curves depicting the relationship between *Kt*/*V*_urea_ values and HRs for CVE. In patients aged <65 years, both unadjusted and adjusted spline curves indicated improved CVE-free survival with high *Kt*/*V*_urea_ values and diminished CVE-free survival with decreasing *Kt*/*V*_urea_ values. However, in other age groups, such a relationship between *Kt*/*V*_urea_ values and CVE was not observed.

### *Kt*/*V*_urea_ and outcomes based on the number of HD sessions per week

The numbers of patients who had two HD sessions per week were 619 (2.3%) in the group aged <65; 365 (3.3%) in the group aged 65–74; 287 (5.0%) in the group aged 75–84; and 43 (7.2%) in that aged ≥8 years (*P *< 0.001). The proportions of patients who had two HD sessions per week were increased as age increased. In patients aged <65 years, the proportion of patients with high *Kt*/*V*_urea_ was significantly greater among those who had three HD sessions per week than among who had two sessions per week (the proportion of high *Kt*/*V*_urea_ in two sessions per week vs. three sessions per week: 56.7% vs. 62.9%) (Table S4). Among patients aged ≥65 years, no significant difference was observed in the proportions of low, ref, and high *Kt*/*V*_urea_ between patients who had two and three HD sessions per week.

In patients who had two HD sessions per week, no significant associations were observed between groups based on *Kt*/*V*_urea_ and mortality or CVEs in both univariable and multivariable analyses (Table S5). The association between *Kt*/*V*_urea_ and mortality in patients who had three HD sessions per week was similar with those in all patients (Table S6). In patients who had three HD sessions per week, multivariable analyses revealed that patients with low *Kt*/*V*_urea_ had highest mortality and those with high *Kt*/*V*_urea_ had the lowest mortality among patients aged <65 years or 65–77 years. Among patients aged 75–84 or ≥85 years, mortality was lower in patients with ref or high *Kt*/*V*_urea_ than in those with low *Kt*/*V*_urea_. In addition, patients with high *Kt*/*V*_urea_ had lower CVE than those with low *Kt*/*V*_urea_ among patients aged <65 years.

### Characteristics and outcomes based on *Kt*/*V*_urea_ in patients aged ≥85 years

We performed further analyses in patients aged ≥85 years (Table S7). The high *Kt*/*V*_urea_ group had fewer male sex representatives and lower body mass index than the other groups, and greater HD vintage than the ref *Kt*/*V*_urea_ group did. In addition, the proportion of diabetic nephropathy was lower in the ref or high *Kt*/*V*_urea_ group than in low *Kt*/*V*_urea_ group, although this difference was not statistically significant. Subgroup analyses were performed based on male sex (female participants were excluded from the analyses because of the small sample size), the presence of diabetic nephropathy, body mass index (based on median 21.6 kg/m^2^), and HD vintage (based on median 26 months). Patient survival rates were greater in the ref or high *Kt*/*V*_urea_ groups than in the low *Kt*/*V*_urea_ group in subgroups of male sex, low body mass index, and long HD vintage (Table S8).

## DISCUSSION

In individuals aged <65 or 65–74 years, survival rates increased with increasing *Kt*/*V*_urea_ values. This trend was consistent in all analyses. In individuals aged 75–84 years, a similar trend was observed but without a significant difference in the survival rate between the ref and high *Kt*/*V*_urea_ groups in both two survival analyses. In individuals aged ≥85 years, both Kaplan–Meier, and Cox regression analyses demonstrated worse patient survival rate in the low *Kt*/*V*_urea_ group over other groups, but the difference in the survival rate between the ref and high *Kt*/*V*_urea_ groups was not noted in all analyses. Overall, the low *Kt*/*V*_urea_ group had lower survival rates than other groups irrespective of age but the improved survival in the high *Kt*/*V*_urea_ group compared with the ref *Kt*/*V*_urea_ group tended to diminish with age.

*Kt*/*V*_urea_ has several limitations. The measurement was developed for a young population with few comorbidities in an era of small-pore bioincompatible dialyzers [9, 10], although several observational studies have since demonstrated an association between *Kt*/*V*_urea_ and survival in various populations [22, 23]. Limited evidence suggests that urea is toxic, and the removal of urea does not effectively reflect the removal of other uremic toxins, especially protein-bound toxins [24]. *Kt*/*V*_urea_ is strongly influenced by any changes in volume (*V*). In small-sized or malnourished patients, *Kt*/*V*_urea_ will overestimate the dose of dialysis and vice versa. Several studies have reported that high *Kt*/*V*_urea_ was associated with high mortality in malnourished patients presumably due to a low volume [25, 26]. Therefore, a need exist to better understand the usefulness of *Kt*/*V*_urea_ in older patients who have a higher comorbidity burden, lower metabolic burden due to decreased calorie and protein intake, and differences in the ratio of urea distribution volume to body weight compared to a younger population [27, 28, 29].

We identified an association between low *Kt*/*V*_urea_ and mortality not only in patients aged 75–84 years but also in those aged ≥85 years using large cohort data, thus adding evidence to support the current guideline recommendations of minimum single-pool *Kt*/*V*_urea_ of 1.2 irrespective of age. One previous study also demonstrated that *Kt*/*V*_urea_ < 1.2 in patients aged ≥75 years is associated with increased mortality [13]. In our cohort, when compared with younger patients, older patients had a lower body mass index and blood levels of albumin, phosphorus, and creatinine, which represent lower protein intake and muscle mass. Furthermore, *V*_urea_ is generally considered equivalent to the total body water. The percentage of body water to body weight decreases in older adults or those with less muscle mass [29, 30]. The Daugirdas equation assumes total body water to be 55% of the body weight irrespective of age; therefore, it overestimates *V*_urea_ in older patients [17]. The recent KDIGO guidelines recommend the use of the Daugirdas equation by calculating body weight from the total estimated body water [7], but whether this method will improve the clinical relevance of *Kt*/*V*_urea_ in older patients remains unclear. The increase in *Kt*/*V*_urea_ with age in our study may be due to decrease in body weight with age. However, *Kt*/*V*_urea_ in older patients can underestimate *Kt*/*V*_urea_ due to an overestimation of the total body water. Despite these limitations, our findings suggest that *Kt*/*V*_urea_ should be maintained above the minimum recommended level even in older patients.

In our study, a higher *Kt*/*V*_urea_ (≥1.4) was linked to lower mortality compared *Kt*/*V*_urea_ 1.2–1.4 in patients aged <65 years. The HEMO study and the international Dialysis Outcomes and Practice Patterns Study did not demonstrate overall survival benefits with high *Kt*/*V*_urea_ [14, 31]. However, unlike the HEMO study in which bioincompatible cellulosic membranes were used in approximately half of the patients, biocompatible synthetic membranes are now used in nearly all patients [32]. Additionally, the overall survival rate of patients with HD has improved since 2000 [3, 33]. Therefore, the optimal HD dose as measured using *Kt*/*V*_urea_ remains controversial. In our study, the HR for mortality decreased with increasing *Kt*/*V*_urea_ in patients aged <85 years. This trend was also observed in recent studies that identified a low risk of mortality with high *Kt*/*V*_urea_ [34, 35, 36]. This trend was not observed in patients aged ≥85 years because high *Kt*/*V*_urea_ may be a sign of malnutrition or wasting conditions in older patients. Further research is needed to determine whether targeting high *Kt*/*V*_urea_ will improve survival in patients on HD, especially in older patients.

Cardiovascular diseases are the main cause of death in patients with ESKD [37]. The association between volume overload or ultrafiltration rate and cardiovascular mortality was well-demonstrated in patients with HD [38, 39]. Our findings revealed that a high *Kt*/*V*_urea_ was associated with a low risk of CVEs in those aged <65 years but not in those aged ≥65 years. In comparison with all-cause mortality, the association between *Kt*/*V*_urea_ and CVEs appears weak, especially in older patients. In these patients, the incidence of cardiovascular complications related to dialysis is often influenced by various factors aimed at achieving an elevated *Kt*/*V*_urea_. Importantly, the pursuit of high *Kt*/*V*_urea_ through increased blood flow rate, ultrafiltration volume, or prolonged HD may ultimately have adverse effects on cardiovascular health in older patients. Consequently, these may be associated with neutral CVE outcomes in older patients despite high *Kt*/*V*_urea_.

Patients who were aged ≥85 years showed poor 5-year survival rate. Previous nationwide cohort study from South Korea also reported similar poor 5-year survival in significantly older patients starting HD [40]. Few studies have reported mortality rates in patients on dialysis who were >85 years of age, while one center in Israel reported a 5-year survival rate of 20% in patients >85 years of age on HD [41]. In addition, the United States Renal Data System also reported a 5-year survival rate of 21.5% in patients aged 75 years and older on HD [42]. Most patients in this age group underwent three HD sessions per week and reached the recommended target *Kt*/*V*_urea_, suggesting that they might have been tolerant to HD procedures. Therefore, we believe that the poor survival in patients aged 85 years and older in our study reflects the shorter life expectancy and higher burden of comorbidities associated with this age group. We conducted subgroup analysis for factors that showed differences at baseline to exclude the effect of other confounding factors in this extremely old group. Patient survival was better in the ref or high *Kt*/*V*_urea_ groups than in low *Kt*/*V*_urea_ group in subgroups of male sex, low body mass index, and long HD vintage. In addition, patients who had three HD sessions per week showed a greater survival in the ref or high *Kt*/*V*_urea_ group than in the low *Kt*/*V*_urea_ group in univariable and multivariable analyses. These results revealed that *Kt*/*V*_urea_ was independently associated with patient survival in very elderly patients. However, in some subgroups, no significant differences were observed in patient survival among three groups, likely due to the limitation caused by the small sample size. Conducting additional studies with larger sample sizes would be helpful to demonstrate the impact of *Kt*/*V*_urea_ on very elderly patients.

Our study has certain limitations. First, it is a retrospective study that analyzed data of patients already on HD. Consequently, drawing clear conclusions about the causal relationship between the two variables is challenging, given several limitations, including potential selection bias indicated by differences in baseline characteristics between groups. In particular, when investigating the relationship between dialysis dose and health outcomes, observational retrospective studies rather than randomized controlled trials have inherent limitations due to the possibility of ‘dose-target bias’ [43]. Poor compliance or inability to achieve target dose may itself influence outcomes. Second, no standardization is present for the measurement of BUN, which is essential for calculating *Kt*/*V*_urea_. This lack of standardization may have resulted in inter-center variability. Third, some important laboratory information was missing, such as pre- and postdialysis BUN levels or protein intake, which can affect the urea generation rate. Consequently, these constitute an important barrier to understanding the independent effect of *Kt*/*V*_urea_. Inflammation and malnutrition can affect urea production and, thus, affect accurate assessments of *Kt*/*V*_urea_. We only used diagnostic codes or procedural information to assess the effects of CVE. Furthermore, our study did not include detailed data on the HD sessions, such as change in blood pressure during HD, HD modalities, and the duration of HD sessions, which could be additional confounding factors of patient outcomes. In addition, our study did not include data on the type of membrane used. However, in South Korea, the use of synthetic membranes has been increasing and already exceeded 90% in 2009 [32]. Fourth, the data in our study only included information regarding all-cause mortality as an outcome with limited information on specific causes of death, such as cachexia, infection, and cardiovascular diseases. Fifth, our analysis was based on *Kt*/*V*_urea_ at the beginning of the follow-up; therefore, it does not reflect changes in *Kt*/*V*_urea_ over time. However, we believe that average *Kt*/*V*_urea_ over a 6-month period reflects the dialysis setting applied on an ongoing basis rather than temporary fluctuations. Sixth, overall, the number of patients in the low *Kt*/*V*_urea_ group was significantly lower than that in the ref and high *Kt*/*V*_urea_ groups, especially in significantly older patients. Therefore, caution is needed when interpreting the results of multivariable analyses. Although definitive conclusions regarding the age-specific association between *Kt*/*V*_urea_ and outcomes may be limited due to the aforementioned factors, this study paves the way for further research in older populations.

In conclusion, *Kt*/*V*_urea_ < 1.2, as recommended by the current guidelines, was associated with poor patient survival irrespective of age. In patients aged <65 or 65–74 years, *Kt*/*V*_urea_ > 1.4 was associated with improved survival; however, the survival benefit was not observed in those aged ≥85 years. Therefore, in young patients, *Kt*/*V*_urea_ over the recommended threshold may be beneficial for survival, and although this trend diminishes with age, it may be still advantageous to achieve the minimum *Kt*/*V*_urea_ suggested by the guidelines, even in older patients aged ≥85 years.

## Supplementary Material

sfae116_Supplemental_File

## Data Availability

The raw data were generated by the Health Insurance Review and Assessment Service. The database can be requested from the Health Insurance Review and Assessment Service by sending a study proposal including the purpose of the study, study design, and duration of analysis through the web site (https://www.hira.or.kr). The authors cannot distribute the data without permission.
