# Supplementary material for: Effects of Kt/Vurea on outcomes according to age in patients on maintenance hemodialysis
Source: Clin Kidney J. 2024 Apr 13;17(5):sfae116. doi: 10.1093/ckj/sfae116 (PMC11099659; doi:10.1093/ckj/sfae116)
Supplement: sfae116_Supplemental_File [file sfae116_supplemental_file.docx]

**Table S1. Medication types and Health Insurance Review and Assessment Service codes**

| **Medications** | **Codes** |
| --- | --- |
| **Alacepril** | 104201ATB, 104202ATB |
| **Benazepril** | 114701ATB |
| **Captopril** | 122901ATB, 122902ATB, 122903ATB |
| **Cilazapril** | 133001ATB, 133002ATB, 133003ATB |
| **Enalapril** | 151601ATB, 151603ATB |
| **Fosinopril** | 163501ATB, 163502ATB |
| **Imidapril** | 173401ATB, 173402ATB |
| **Moexipril** | 196801ATB, 196802ATB |
| **Lisinopril** | 184501ATB |
| **Perindopril** | 211301ATB, 211302ATB, 501601ATB, 501602ATB |
| **Quinapril** | 221901ATB, |
| **Ramipril** | 222401ATB, 222402ATB, 222404ATB |
| **Zofenopril** | 510401ATB, 510402ATB, 510403ATB |
| **Temocapril** | 235002ATB |
| **Delapril** | 140901ATB, 140902ATB |
| **Captopril + Hydrochlorothiazide** | 262200ATB, 262300ATB |
| **Enalapril + Hydrochlorothiazide** | 440300ATB, 453700ATB, 453600ATB |
| **Ramipril + Felodipine** | 447100ATB, 447200ATB |
| **Ramipril + Hydrochlorothiazide** | 448600ATB, 448700ATB |
| **Perindopril + indapamide** | 556200ATB |
| **Lisinopril + Hydrochlorothiazide** | 499200ATB, 499300ATB |
| **Moexipril + Hydrochlorothiazide** | 440800ATB, 497900ATB |
| **Enalapril + nitrendipine** | 466000ATB |
| **Candesartan** | 122601ATB, 122602ATB, 122603ATB |
| **Irbesartan** | 177301ATB, 177303ATB |
| **Losartan** | 185701ATB, 185702ATB |
| **Valsartan** | 247101ATB, 247102ATB, 247103ATB, 247104ATB |
| **Fimasartan** | 515201ATB, 515202ATB, 515203ATB |
| **Azilsartan** | 662401ATB, 662402ATB, 662403ATB |
| **Telmisartan** | 378801ATB, 378802ATB |
| **Eprosartan** | 429201ATB |
| **Olmesartan** | 468501ATB, 468502ATB, 468503ATB, 520901ATB, 520902ATB |
| **Valsartan + Amlodipine** | 492800ATB, 492900ATB, 495800ATB, 522600ABTB, 522700ABTB, 522800ABTB, 522900ABTB, 523000ATB, 523100ATB, 523200ATB, 523300ATB, 523400ATB |
| **Valsartan + Lercanidipne** | 522200ATB. 522300ATB. 522400ATB |
| **Valsartan + Pitavastatin** | 634900ATB, 635000ATB, 635100ATB, 635200ATB |
| **Valsartan + Sacubitril** | 651401ATB, 651402ATB, 651403ATB |
| **Valsartan + Rosuvastatin** | 629700ATB, 629800ATB, 525000ATB, 525100ATB, 525200ATB, 525300ATB, |
| **Valsartan + Hydrochlorothiazide** | 356400ATB, 442600ATB |
| **Olmesaetan + Amlodipine** | 500500ATB, 500600ATB, 547500ATB. 547600ATB, 547700ATB, 547800ATB, 547900ATB, 548000ATB, 582200ATB, 582400ATB, 629400ATB, 629500ATB, 629600ATB, 631300ATB, 632800ATB, 632900ATB, 633000ATB |
| **Olmesartan + Hydrochlorothiazide** | 513600ATB |
| **Olmesartan + Hydrochlorothiazide + Amlodipine** | 519700ATB, 519800ATB, 519900ATB, 520000ATB, 520100ATB |
| **Olmesartan + Rosuvastatin** | 653200ATB, 644100ATB, 644200ATB, 526300ATB, 526400ATB, 526500ATB, 526900ATB |
| **Telmisartan + Hydrochlorothiazide** | 502600ATB, 443200ATB, 443300ATB |
| **Telmisartan + Rosuvastatin** | 629900ATB, 630000ATB, 630100ATB, 630200ATB, 631600ATB, 631700ATB |
| **Telmisartan + Amlodipine** | 511500ATB, 511600ATB, 511700ATB, 521200ATB, 521300ATB, 521400ATB, 623100ATB, 644800ATB |
| **Telmisartan+ Hydrochlorothiazide + Amlodipine** | 663500ATB, 663600ATB, 663700ATB, 663800ATB |
| **Telmisartan + Rosuvastatin + Amlodipine** | 671700ATB, 671600ATB, 671500ATB, 671400ATB, 671300ATB, 671200ATB, |
| **Losartan + Hydrochlorothiazide** | 262500ATB, 378900ATB, 486900ATB |
| **Losartan + Amlodipine** | 502700ATB, 503000ATB, 513900ATB, 637400ATB, 637500ATB, 637600ATB |
| **Losa+rsvt+ Amlodipine** | 663900ATB, 664000ATB, 664100ATB, 664200ATB, 664300ATB, 664400ATB, |
| **Losa+chlor+ Amlodipine** | 662800ATB, 662900ATB, 663000ATB |
| **Fimasartan + Hydrochlorothiazide** | 522000ATB, 526800ATB |
| **Fimasartan + Amlodipine** | 651900ATB, 652000ATB, 652100ATB, 652700ATB, 651900ATB |
| **Fimasartan + Rosuvastatin** | 654600ATB, 654700ATB, 654800ATB, 654900ATB, 655000ATB |
| **Candesartan + Hydrochlorothiazide** | 423700ATB |
| **Candesartan + Amlodipine** | 652900ATB, 653000ATB, 653100ATB, 652900ATB, 652900ATB |
| **Candesartan + Rosuvastatin** | 673700ATB, 661800ATB, 661900ATB, 662000ATB, 662100ATB |
| **Irbesartan + Hydrochlorothiazide** | 385700ATB, 385800ATB |
| **Irbesartan + Atorvastatin** | 527000ATB, 527100ATB, 524000ATB, 524100ATB |
| **Azilsartan + Chlorthalidone** | 673500ATB, 673600ATB |
| **Eprosartan + Hydrochlorothiazide** | 460500ATB |
| **Amlodipine** | 495901ATB, 459802ACH, 483201ATB, 486501ATB, 107601ATB, 107601ATD, 459801ACH, 459801ATB, 459901ATB, 464601ATB, 470801ATB, 476201ATB, 479701ATB, 483202ATB, 486502ATB, 107602ATB, 107602ATD, 470802ATB |
| **Amlodipine + Atorvastatin** | 614500ATB, 472300ATB, 472400ATB, 472500ATB, 518900ATB |
| **Amlodipine + Rosuvastatin** | 673900ATB, 674000ATB, 674100ATB |
| **Amosulalol** | 107901ATB, 107902ATB |
| **Arotinolol** | 110202ATB, 110201ATB |
| **Atenolol** | 483102ATB, 111402ATB, 483101ATB, 111403ATB, 111401ATB |
| **Atenolol + Chlorthalidone** | 262100ATB, 460200ATB |
| **Barnidipine** | 114003ACH, 114001ACH, 114002ACH |
| **Benidipine** | 115101ATB, 115102ATB, 115104ATB, 115103ATB |
| **Betaxolol** | 116801ATB, 116803ATB |
| **Bevantolol** | 117002ATB, 117001ATB |
| **Bisoprolol** | 117904ATB, 117903ATB, 117902ATB, 117901ATB |
| **Bisoprolol + Hydrochlorothiazide** | 469800ATB, 470000ATB, 469900ATB |
| **Carteolol** | 124801ATB |
| **Carvedilol** | 125005ATB, 125003ATB, 662201ATB, 125008ACR, 125001ATB, 662202ATB, 125007ACR, 125002ATB, 125006ACR, 125004ACR |
| **Celiprolol** | 129101ATB |
| **Cilnidipine** | 133102ATB, 133101ATB |
| **Clonidine** | 136505ATR |
| **Diltiazem** | 145706ATB, 145707ACR, 145707ATR, 145703ACR, 145706ATR, 145707ATB |
| **Doxazocin** | 149101ATB, 149102ATB, 149104ATR, 149103ATB |
| **Efonidipine** | 441202ATB, 441201ATB |
| **Felodipine** | 157503ATR, 157501ATR |
| **Felodipine + Metoprolol** | 262400ATR |
| **Hydralazine** | 170701ATB |
| **Lacidipine** | 180301ATB, 180302ATB, 180303ATB |
| **Lercanidipine** | 182001ATB, 182002ATB |
| **Manidipine** | 188001ATB, 188002ATB |
| **Metoprolol** | 194003ATR, 193802ATB, 262400ATR |
| **Metoprolol + Hydrochlorothiazide** | 262600ATB |
| **Metoprolol + Felodipine** | 262400ATR |
| **Minoxidil** | 196102ATB |
| **Nadolol** | 198301ATB |
| **Nebivolol** | 489501ATB, 489502ATB, 489503ATB |
| **Nicardipine** | 201003ACR, 201002ATB |
| **Nifedipine** | 201407ACS, 201405ATR, 528201ATR, 201409ATR, 528202ATR, 201401ACS, 201401ATB, 201408ATR |
| **Nimodipine** | 201901ATB, 356202ATR, 356203ATR, 356201ATB, 356202ATB |
| **Nisoldipine** | 356202ATR |
| **Propranolol** | 219901ATB, 219904ATB, 219906ACR, 219905ACR |
| **Terazosin** | 235501ATB, 235502ATB, 235503ATB, 616501ATB |
| **Verapamil** | 247606ATB, 247607ATB, 247603ATR, 247605ATR, 247601ACR |
| **Atorvastatin + Ezetimibe** | 633800ATB, 633900ATB, 634800ATB |
| **Pitavastatin + Fenofibrate** | 679300ACH |
| **Rosuvastatin + Ezetimibe** | 640700ATB, 640800ATB, 640900ATB |
| **Aspirin** | 110701ATB, 110702ATB, 110801ATB, 110802ATB, 111001ACE, 111001ATB, 111001ATE, 111002ATE, 111003ACE, 111003ATE |
| **Clopidogrel** | 133201ACR, 133201ATB, 133201ATR, 133202ATB, 133203ATR, 506100ATB |
| **Cilostazol** | 136901ATB, 492501ATB, 495201ATB, 498801ATB, 501501ATB |
| **Ticlopidine** | 498900ATB, 239201ATB, 239202ATB |
| **Aspirin + Bethocarbamol** | 256800ATB |
| **Aspirin + Clopidogrel** | 517900ACH, 517900ACE, 517900ATE, 667500ACE |
| **Aspirin + Dipyridamole** | 489700ACR |
| **Atorvastatin** | 111502ATB, 502202ATB, 633900ATB, 472400ATB, 518900ATB, 524100ATB, 527000ATB, 672000ATR, 672100ATR, 111503ATB, 502203ATB, 634800ATB, 472500ATB, 111504ATB, 502204ATB |
| **Fluvastatin** | 162401ACH, 162402ACH, 162403ATR |
| **Lovastatin** | 185801ATB |
| **Pitavastatin** | 470901ATB, 470902ATB, 470903ATB |
| **Pravastatin** | 216601ATB, 216602ATB, 216603ATB, 216604ATB |
| **Rosuvastatin** | 454001ATB, 454002ATD, 454002ATB, 454003ATB, 454003ATD, 454005ATB |
| **Simvastatin** | 227801ATB, 227802ATB, 227803ATB, 227805ATB, 227806ATB |

**Table S2. ICD–10 codes used in the Charlson Comorbidity Index**

| **Comorbidities** | **Codes** | **Score** |
| --- | --- | --- |
| Myocardial infarction | I21, I22, I252 | 1 |
| Congestive heart failure | I43, I50, I099, I110, I130, I132, I255, I420, I425–I429, P290 | 1 |
| Peripheral vascular disease | I70, I71, I731, I738, I739, I771, I790, I792, K551, K558, K559, Z958, Z959 | 1 |
| Cerebrovascular disease | G45, G46, I60–69, H340 | 1 |
| Dementia | F00–03, G30, F051, G311 | 1 |
| Chronic pulmonary disease | J40–47, J60–67, I278–279, J701, J703, J684 | 1 |
| Rheumatologic disease | M05–06, M32–34, M315, M351, M353, M360 | 1 |
| Peptic ulcer disease | K25–28 | 1 |
| Mild liver disease | B18, K73, 74, K700–703, K709, K713–715, K717, K760, K762–764, K768–769, Z944 | 1 |
| DM without complication | E100–101, E106, E108–111, E116, E118–121, E126, E128–131, E136, E138–141, E146, E148–149 | 1 |
| DM with complication | E102–105, E107, E112–115, E117, E122–125, E127, E132–135, E137, E142–145, E147 | 2 |
| Hemiplegia or paraplegia | G81–82, G041, G114, G800, G830–834, G839 | 2 |
| Any malignancy | C00–26, C30–C34, C37–41, C43, C45–58, C60–6, C81–88, C90–97 | 2 |
| Moderate to severe liver disease | I850, I859, I864, I982, K704, K711, K721, K729, K765–767 | 3 |
| Metastatic tumor | C77–80 | 6 |
| AIDS/HIV | B20–22, B24. | 6 |

Abbreviations: ICD–10, International Classification of Diseases, 10th revision, Clinical Modification; DM, diabetes mellitus; AIDS/HIV, acquired immune deficiency syndrome/human immunodeficiency virus

**Table S3. Definitions and codes for cardiovascular events.**

| **ICD–10 codes** |  |
| --- | --- |
| Myocardial infarction | I21–23 |
| Stroke | I60–63 |
| **Procedure or operation codes** |  |
| Percutaneous coronary intervention | M6551–2, M6561–4, M6571–2, M6601–2 |
| Coronary artery bypass grafting | O1641–2, O1647, OA641–2, OA647 |
| **Medical treatment codes** |  |
| Protein C | 635801BIJ |
| Tissue type plasminogen activator | 223501BIJ–2BIJ |
| Tenecteplase | 450301BIJ–2BIJ |
| Tirofiban | 240201BIJ, 240230BIJ |
| Urokinase | 246401BIJ, 246404BIJ–407BIJ |

**Table S4. Proportions of patients based on the number of hemodialysis sessions per week**

|  | **< 65 years** | | | **65–74 years** | | | **75–84 years** | | | **≥ 85 years** | | |
| --- | --- | --- | --- | --- | --- | --- | --- | --- | --- | --- | --- | --- |
|  | **Two** | **Three** | ***P*** | **Two** | **Three** | ***P*** | **Two** | **Three** | ***P*** | **Two** | **Three** | ***P*** |
| **Ref** | 213 (34.4%) | 7,943 (30.8%) | 0.002 | 113  (31.0%) | 2,945  (27.2%) | 0.108 | 64 (22.3%) | 1,298  (23.6%) | 0.098 | 9  (20.9%) | 117  (21.2%) | 0.568 |
| **Low** | 55  (8.9%) | 1,613  (6.3%) |  | 20  (5.5%) | 454  (4.2%) |  | 18  (6.3%) | 207  (3.8%) |  | 0 | 14  (2.5%) |  |
| **High** | 351  (56.7%) | 16,195  (62.9%) |  | 232  (63.6%) | 7,414  (68.6%) |  | 205  (71.4%) | 3,989  (72.6%) |  | 34  (79.1%) | 421  (76.3%) |  |

Ref, patients with 1.2 ≤ Kt/V_urea_ ≤ 1.4; Low group, patients with Kt/V_urea_ < 1.2; High group, patients with Kt/V_urea_ > 1.4. *P*–values are tested using a Pearson’s χ^2^ test.

**Table S5.** **Kt/V_urea_ and the associated risk of all-cause mortality and cardiovascular events in patients who have two hemodialysis sessions per week**

|  | **All-cause mortality** | | | | **Cardiovascular events** | | | |
| --- | --- | --- | --- | --- | --- | --- | --- | --- |
|  | **Univariable** | | **Multivariable** | | **Univariable** | | **Multivariable** | |
|  | **HR (95% CI)** | ***P*** | **HR (95% CI)** | ***P*** | **HR (95% CI)** | ***P*** | **HR (95% CI)** | ***P*** |
| **< 65 years** |  |  |  |  |  |  |  |  |
| Reference: Ref |  |  |  |  |  |  |  |  |
| Low | 0.68 (0.34–1.33) | 0.256 | 0.76 (0.38–1.53) | 0.440 | 0.96 (0.41–2.20) | 0.914 | 1.22 (0.51–2.88) | 0.657 |
| High | 0.98 (0.69–1.39) | 0.901 | 1.34 (0.89–2.02) | 0.166 | 0.96 (0.59–1.57) | 0.873 | 1.33 (0.74–2.39) | 0.344 |
| Reference: Low |  |  |  |  |  |  |  |  |
| High | 1.45 (0.75–2.80) | 0.271 | 1.76 (0.86–3.61) | 0.123 | 1.01 (0.45–2.24) | 0.988 | 1.09 (0.45–2.67) | 0.847 |
| **65–74 years** |  |  |  |  |  |  |  |  |
| Reference: Ref |  |  |  |  |  |  |  |  |
| Low | 0.73 (0.33–1.61) | 0.433 | 0.66 (0.28–1.59) | 0.359 | 0.67 (0.20–2.24) | 0.517 | 0.87 (0.24–3.09) | 0.823 |
| High | 1.07 (0.76–1.51) | 0.696 | 1.15 (0.78–1.70) | 0.472 | 0.82 (0.49–1.39) | 0.464 | 1.11 (0.59–2.09) | 0.756 |
| Reference: Low |  |  |  |  |  |  |  |  |
| High | 1.47 (0.68–3.17) | 0.323 | 1.74 (0.73–4.11) | 0.210 | 1.23 (0.38–3.97) | 0.735 | 1.28 (0.36–4.58) | 0.706 |
| **75–84 years** |  |  |  |  |  |  |  |  |
| Reference: Ref |  |  |  |  |  |  |  |  |
| Low | 0.79 (0.41–1.53) | 0.484 | 0.70 (0.35–1.42) | 0.324 | 2.38 (0.85–6.69) | 0.100 | 2.80 (0.96–8.19) | 0.060 |
| High | 0.89 (0.63–1.27) | 0.537 | 0.80 (0.54–1.19) | 0.273 | 1.23 (0.59–2.55) | 0.579 | 1.18 (0.52–2.69) | 0.690 |
| Reference: Low |  |  |  |  |  |  |  |  |
| High | 1.13 (0.61–2.11) | 0.689 | 1.27 (0.65–2.47) | 0.484 | 0.52 (0.22–1.22) | 0.133 | 0.42 (0.16–1.09) | 0.074 |
| **≥ 85 years** |  |  |  |  |  |  |  |  |
| Reference: Ref |  |  |  |  |  |  |  |  |
| High | 1.18 (0.49–2.85) | 0.710 | 1.59 (0.23–10.87) | 0.636 | 3.93 (0.51–30.04) | 0.188 | 2.34 (0.04–133.9) | 0.680 |

Multivariable analysis is adjusted for age, sex, body mass index, vascular access type, hemodialysis vintage, Charlson Comorbidity Index score, ultrafiltration volume, hemoglobin, serum albumin, serum creatinine, serum phosphorus, serum calcium, use of anti-hypertensive drugs, statin, clopidogrel, or aspirin, and myocardial infarction or congestive heart failure, and is performed using enter mode. Ref group: patients with 1.2 ≤ Kt/V_urea_ ≤ 1.4; Low group, patients with Kt/V_urea_ < 1.2; High group, patients with Kt/V_urea_ > 1.4.

**Abbreviations**: HR, hazard ratio; CI, confidence interval.

**Table S6.** **Kt/V_urea_ and the associated risk of all-cause mortality and cardiovascular events in patients who have three hemodialysis sessions per week**

|  | **All-cause mortality** | | | | **Cardiovascular events** | | | |
| --- | --- | --- | --- | --- | --- | --- | --- | --- |
|  | **Univariable** | | **Multivariable** | | **Univariable** | | **Multivariable** | |
|  | **HR (95% CI)** | ***P*** | **HR (95% CI)** | ***P*** | **HR (95% CI)** | ***P*** | **HR (95% CI)** | ***P*** |
| **< 65 years** |  |  |  |  |  |  |  |  |
| Reference: Ref |  |  |  |  |  |  |  |  |
| Low | 1.17 (1.06–1.29) | 0.003 | 1.25 (1.13–1.39) | <0.001 | 1.10 (0.96–1.26) | 0.170 | 1.11 (0.96–1.28) | 0.143 |
| High | 0.86 (0.82–0.91) | <0.001 | 0.86 (0.81–0.91) | <0.001 | 0.90 (0.84–0.97) | 0.006 | 0.95 (0.87–1.03) | 0.173 |
| Reference: Low |  |  |  |  |  |  |  |  |
| High | 0.74 (0.67–0.81) | <0.001 | 0.69 (0.62–0.76) | <0.001 | 0.82 (0.72–0.94) | 0.003 | 0.85 (0.74–0.98) | 0.024 |
| **65–74 years** |  |  |  |  |  |  |  |  |
| Reference: Ref |  |  |  |  |  |  |  |  |
| Low | 1.10 (0.97–1.25) | 0.147 | 1.16 (1.02–1.32) | 0.028 | 1.03 (0.82–1.29) | 0.814 | 1.01 (0.80–1.27) | 0.943 |
| High | 0.91 (0.86–0.96) | 0.001 | 0.92 (0.87–0.99) | 0.016 | 0.94 (0.85–1.04) | 0.223 | 1.03 (0.92–1.15) | 0.657 |
| Reference: Low |  |  |  |  |  |  |  |  |
| High | 0.83 (0.74–0.94) | 0.003 | 0.80 (0.70–0.91) | <0.001 | 0.92 (0.74–1.14) | 0.422 | 1.02 (0.81–1.28) | 0.888 |
| **75–84 years** |  |  |  |  |  |  |  |  |
| Reference: Ref |  |  |  |  |  |  |  |  |
| Low | 1.33 (1.13–1.58) | <0.001 | 1.38 (1.16–1.63) | <0.001 | 0.93 (0.64–1.36) | 0.715 | 0.91 (0.61–1.35) | 0.645 |
| High | 1.03 (0.95–1.11) | 0.526 | 0.99 (0.91–1.08) | 0.882 | 0.96 (0.82–1.12) | 0.607 | 0.98 (0.83–1.16) | 0.837 |
| Reference: Low |  |  |  |  |  |  |  |  |
| High | 0.77 (0.66–0.90) | 0.001 | 0.72 (0.61–0.85) | <0.001 | 1.03 (0.72–1.49) | 0.869 | 1.08 (0.73–1.59) | 0.702 |
| **≥ 85 years** |  |  |  |  |  |  |  |  |
| Reference: Ref |  |  |  |  |  |  |  |  |
| Low | 1.93 (1.08–3.44) | 0.027 | 2.15 (1.18–3.90) | 0.012 | 0.77 (0.10–5.86) | 0.797 | 1.00 (0.13–7.94) | 0.999 |
| High | 1.00 (0.80–1.25) | 0.988 | 0.93 (0.73–1.18) | 0.542 | 1.26 (0.69–2.29) | 0.460 | 1.36 (0.69–2.67) | 0.374 |
| Reference: Low |  |  |  |  |  |  |  |  |
| High | 0.52 (0.30–0.90) | 0.021 | 0.43 (0.24–0.77) | 0.005 | 1.64 (0.23–11.87) | 0.624 | 1.36 (0.18–10.29) | 0.767 |

Multivariable analysis is adjusted for age, sex, body mass index, vascular access type, hemodialysis vintage, Charlson Comorbidity Index score, ultrafiltration volume, hemoglobin, serum albumin, serum creatinine, serum phosphorus, serum calcium, use of anti-hypertensive drugs, statin, clopidogrel, or aspirin, and myocardial infarction or congestive heart failure, and is performed using enter mode. Ref group: patients with 1.2 ≤ Kt/V_urea_ ≤ 1.4; Low group, patients with Kt/V_urea_ < 1.2; High group, patients with Kt/V_urea_ > 1.4.

**Abbreviations**: HR, hazard ratio; CI, confidence interval.

**Table S7. Clinical characteristics by groups based on Kt/V_urea_ in patients aged ≥ 85 years**

|  | **Ref (n = 126)** | **Low (n = 14)** | **High (n = 455)** | ***P*** |
| --- | --- | --- | --- | --- |
| Age (years) | 87.1 ± 2.5 | 88.4 ± 2.8 | 86.9 ± 2.2 | 0.064 |
| Sex (male, %) | 92 (73.0%) | 12 (85.7%) | 210 (46.2%) | <0.001 |
| Hemodialysis vintage (months) | 31 ± 29 | 26 ± 18 | 42 ± 40^*^ | 0.005 |
| Body mass index (kg/m^2^) | 22.8 ± 3.1 | 23.3 ± 3.7 | 21.4 ± 2.9^*#^ | <0.001 |
| Underlying causes of ESKD |  |  |  | 0.113 |
| Diabetes mellitus | 51 (40.5%) | 9 (64.3%) | 152 (33.4%) |  |
| Hypertension | 45 (35.7%) | 3 (21.4%) | 190 (41.8%) |  |
| Glomerulonephritis | 1 (0.8%) | 0 | 21 (4.6%) |  |
| Others | 12 (9.5%) | 0 | 34 (7.5%) |  |
| Unknown | 17 (13.5%) | 2 (14.3%) | 58 (12.7%) |  |
| CCI score | 8.6 ± 2.7 | 9.1 ± 3.1 | 8.4 ± 2.8 | 0.460 |
| Autologous arteriovenous fistula | 100 (70.4%) | 7 (50%) | 321 (70.5%) | 0.027 |
| Kt/V_urea_ | 1.31 ± 0.06 | 1.17 ± 0.03^*^ | 1.67 ± 0.18^*#^ | <0.001 |
| Ultrafiltration volume (L/session) | 1.66 ± 0.93 | 1.68 ± 0.83 | 1.71 ± 0.78 | 0.800 |
| Hemoglobin (g/dL) | 10.5 ± 0.7 | 10.4 ± 0.7 | 10.6 ± 0.6 | 0.389 |
| Serum albumin (g/dL) | 3.78 ± 0.31 | 3.87 ± 0.38 | 3.75 ± 0.35 | 0.267 |
| Serum phosphorus (mg/dL) | 4.22 ± 1.12 | 4.03 ± 0.70 | 4.14 ± 1.00 | 0.630 |
| Serum calcium (mg/dL) | 8.71 ± 0.61 | 8.68 ± 0.52 | 8.73 ± 0.79 | 0.948 |
| Serum creatinine (mg/dL) | 6.9 ± 2.1 | 7.2 ± 2.2 | 7.0 ± 2.0 | 0.845 |
| Use of anti-hypertensive drugs | 70 (55.6%) | 6 (42.9%) | 280 (61.5%) | 0.203 |
| Use of aspirin | 50 (39.7%) | 6 (42.9%) | 208 (45.7%) | 0.480 |
| Use of clopidogrel | 18 (14.3%) | 2 (14.3%) | 90 (19.8%) | 0.342 |
| Use of statins | 29 (23.0%) | 2 (14.3%) | 146 (32.1%) | 0.063 |
| MI or CHF | 66 (52.4%) | 7 (50.0%) | 271 (59.6%) | 0.295 |

Data are expressed as mean ± standard deviation for continuous variables and numbers (percentages) for categorical variables. *P*-values are tested using a one-way analysis of variance, followed by Tukey post-hoc test, and Pearson’s χ^2^ test for categorical variables.

Ref, patients with 1.2 ≤ Kt/V_urea_ ≤ 1.4; Low, patients with Kt/V_urea_ < 1.2; High, patients with Kt/V_urea_ > 1.4.

**Abbreviations:** CCI, Charlson Comorbidity Index; CHF, congestive heart failure; ESKD, end-stage kidney disease; MI, myocardial infarction. ^*^*P* < 0.05 vs. Ref; ^#^*P* < 0.05 vs. Low.

**Table S8. Patient and cardiovascular events free survival rates in subgroups within patients aged ≥ 85 years**

|  | **Patient survival rate at 2 years** | | |  | | **CVE-free survival rate at 2 years** | | | |
| --- | --- | --- | --- | --- | --- | --- | --- | --- | --- |
|  | **Ref** | **Low** | **High** | |  | | **Ref** | **Low** | **High** |
| Male sex | 69.6% | 41.7%* | 66.7%^#^ | |  | | 90.5% | 87.5% | 89.0% |
| Diabetes | 64.7% | 55.6% | 65.1% | |  | | 88.7% | 100% | 83.9% |
| Non-diabetes | 69.3% | 40.0% | 66.0% | |  | | 94.3% | 66.7% | 89.6% |
| BMI < 21.6 kg/m^2^ | 71.7% | 20.0%* | 59.8%^#^ | |  | | 90.9% | 66.7% | 89.2% |
| BMI ≥ 21.6 kg/m^2^ | 65.0% | 66.7% | 73.0% | |  | | 92.7% | 100% | 86.2% |
| HD vintage < 26M | 72.6% | 85.7% | 72.7% | |  | | 92.5% | 100% | 87.9% |
| HD vintage ≥ 26M | 60.0% | 14.3%* | 59.2%^#^ | |  | | 90.8% | 75.0% | 88.0% |

Statistical analysis is performed using Log-rank test of Kaplan–Meier method. **P* < 0.05 vs. Ref group; ^#^*P* < 0.05 vs. Low group.

Ref, patients with 1.2 ≤ Kt/V_urea_ ≤ 1.4; Low group, patients with Kt/V_urea_ < 1.2; High group, patients with Kt/V_urea_ > 1.4.

**Abbreviations:** BMI, body mass index; CVE, cardiovascular event; HD, hemodialysis; M, months.

**
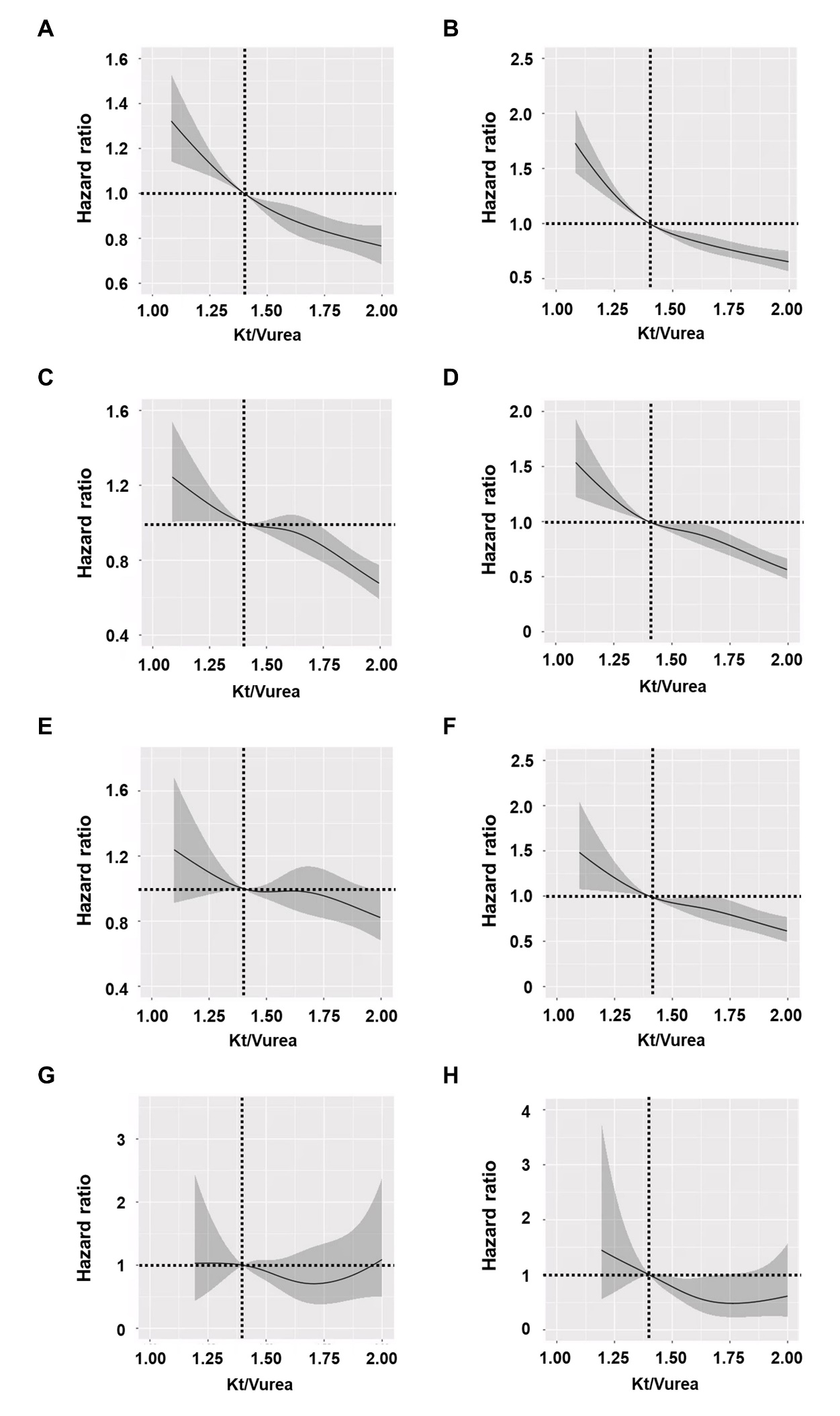

Figure S1. Restrictive spline curves displaying unadjusted and adjusted hazard ratios for all–cause mortality.** Spline curves are expressed in patients with age < 65 years (A, univariable; B, multivariable), 65–74 years (C, univariable; D, multivariable), 75–84 years (E, univariable; F, multivariable), or ≥ 85 years (G, univariable; H, multivariable).

The model is plotted as restricted cubic splines with four knots and the multivariable model is adjusted for age, sex, body mass index, vascular access type, hemodialysis vintage, Charlson Comorbidity Index score, ultrafiltration volume, blood hemoglobin, serum albumin, serum creatinine, serum phosphorus, serum calcium, use of anti–hypertensive drug, statin, clopidogrel, or aspirin, and myocardial infarction or congestive heart failure. The dotted line displays the hazard ratio with 1.4 as the reference value for Kt/V_urea_.

**
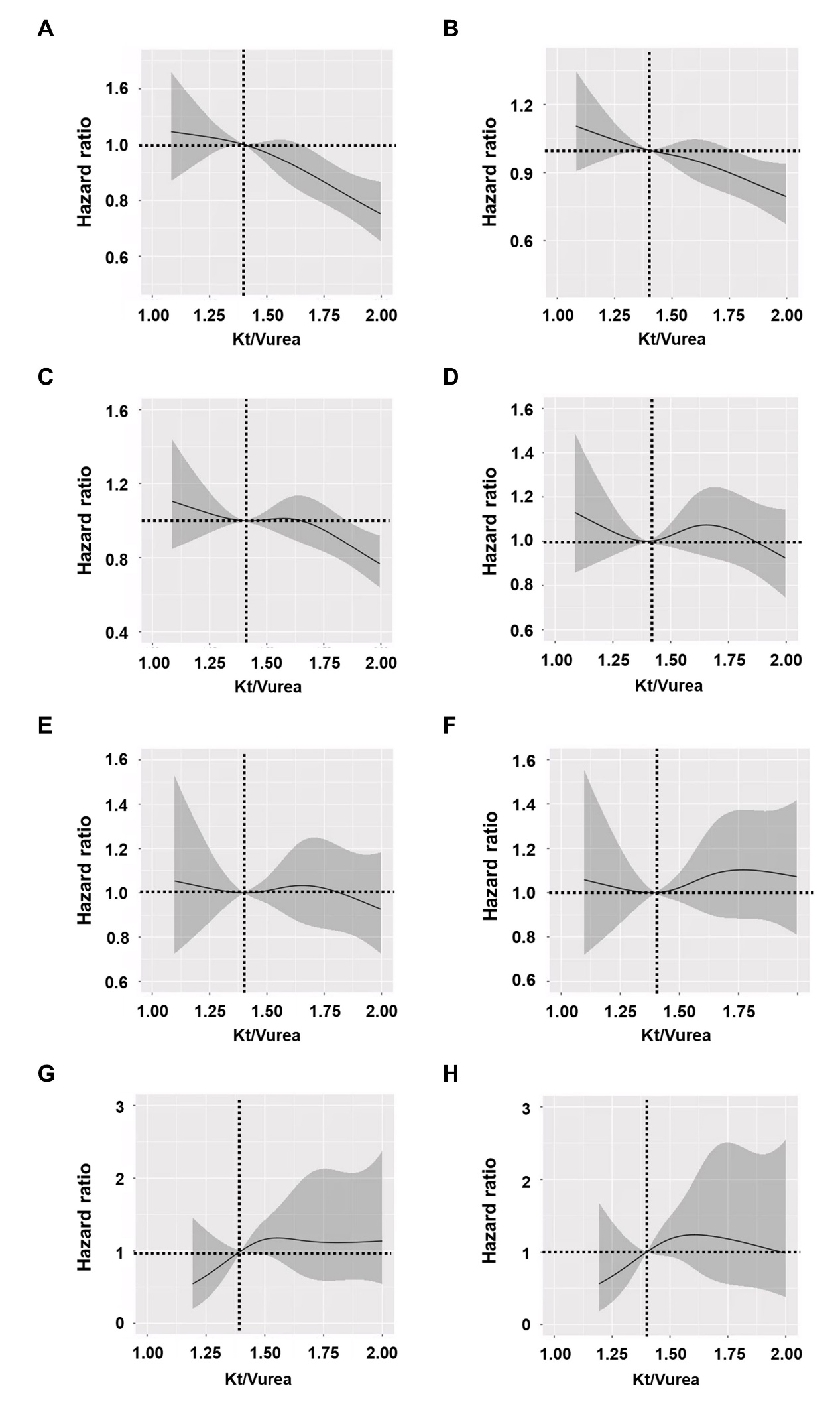
**

**Figure S2. Restrictive spline curve displaying unadjusted and adjusted hazard ratios for cardiovascular events.** Spline curves are expressed in patients with age < 65 years (A, univariable; B, multivariable), 65–74 years (C, univariable; D, multivariable), 75–84 years (E, univariable; F, multivariable), or ≥ 85 years (G, univariable; H, multivariable).

The model is plotted as restricted cubic splines with four knots and the multivariable model is adjusted for age, sex, body mass index, vascular access type, hemodialysis vintage, Charlson Comorbidity Index score, ultrafiltration volume, blood hemoglobin, serum albumin, serum creatinine, serum phosphorus, serum calcium, use of anti–hypertensive drug, statin, clopidogrel, or aspirin, and myocardial infarction or congestive heart failure. The dotted line displays the hazard ratio with 1.4 as the reference value for Kt/V_urea_.
